# Hedgehog pathway activation in human transitional cell carcinoma of the bladder

**DOI:** 10.1038/bjc.2012.55

**Published:** 2012-02-23

**Authors:** G Pignot, A Vieillefond, S Vacher, M Zerbib, B Debre, R Lidereau, D Amsellem-Ouazana, I Bieche

**Affiliations:** 1 Department of Urology, Service d’Urologie, Université Paris Descartes, Sorbonne Paris Cité, 27 rue du Faubourg Saint Jaques, Paris F-75014, France; 2 Department of Urology, Hôpital Cochin, AP-HP, Paris F-75014, France; 3 Department of Pathology, Hôpital Cochin, AP-HP, Paris F-75014, France; 4 Laboratoire d’Oncogénétique – Institut Curie – Hôpital René Huguenin, Saint-Cloud F-92211, France

**Keywords:** bladder cancer, Hedgehog signalling pathway, molecular marker, prognostic factor, RT–PCR

## Abstract

**Background:**

The Hedgehog (Hh) signalling pathway functions as an organiser in embryonic development. Recent studies have shown constitutive activation of this pathway in various malignancies, but its role in bladder cancer remains poorly studied.

**Methods:**

Expression levels of 31 genes and 9 microRNAs (miRNAs) involved in the Hh pathway were determined by quantitative real-time RT–PCR in 71 bladder tumour samples (21 muscle-invasive (MIBC) and 50 non-muscle-invasive (NMIBC) bladder cancers), as well as in 6 bladder cancer cell lines.

**Results:**

The *SHH* ligand gene and Gli-inducible target genes (*FOXM1, IGF2, OSF2, H19, and SPP1)* were overexpressed in tumour samples as compared with normal bladder tissue. *SHH* overexpression was found in 96% of NMIBC and 52% of MIBC samples, as well as in two bladder cancer cell lines. Altered expression of miRNAs supported their oncogene or tumour-suppressor gene status. In univariate analysis, high expression levels of *PTCH2*, miRNA-92A, miRNA-19A, and miRNA-20A were associated with poorer overall survival in MIBC (*P*=0.02, *P*=0.012, *P*=0.047, and *P*=0.036, respectively).

**Conclusion:**

We observed constitutive activation of the Hh pathway in most NMIBC and about 50% of MIBC. We also found that some protein-coding genes and miRNAs involved in the Hh pathway may have prognostic value at the individual level.

In western countries, bladder cancer is the fourth and ninth most common malignancy in men and women, respectively. About 90% of malignancies arising in the urothelium are of epithelial origin (transitional cell carcinoma, TCC). About two-thirds of newly diagnosed cases of TCC are superficial papillary tumours, which are frequently recurrent. The TCC is muscle-invasive at diagnosis in about one-third of cases and metastatic in about 7% of cases. Patients with a given tumour stage and grade may have different outcomes, which cannot be predicted by current prognostic factors, namely TNM stage and pathological grade. New prognostic molecular markers, which might also serve as therapeutic targets, are therefore needed.

The Hedgehog (Hh) family of proteins regulates a wide variety of developmental processes, and Hh pathway defects have been implicated in many developmental disorders (Ingham and McMahon, 2001). However, continuous Hh pathway activity has a role in the growth of various malignancies, that together account for approximately one-quarter of all cancer deaths (Berman *et al*, 2003; Pasca di Magliano and Hebrok, 2003; Scott, 2003; Beachy *et al*, 2004; Fan *et al*, 2004; Kayed *et al*, 2004; Liu *et al*, 2007).

Three *Hh* genes have been described in mammals: Sonic (*SHH*), Indian (*IHH*), and Desert (*DHH*; McMahon, 2000; Nybakken and Perrimon, 2002). The Hh proteins are ligands for the patched receptor (Ptch), which negatively regulates smoothened protein (Smo). The Ptch binds to the Hh proteins, resulting in Ptch internalisation in endosomes and lifting Ptch-mediated repression. This allows Smo to move from an intracellular compartment to the cell surface, resulting in Smo activation and signal transmission. Two homologous Ptch receptors, Ptch1 and Ptch2, have been described, both of which are able to interact with the Hh ligands and Smo protein. Downstream of Smo, the Hh signal activates target genes through the Gli family of zinc-finger transcription factors (including Gli1, Gli2, Gli3, and Gli4 in vertebrates). The *Ptch1* is also a target of this pathway, forming a negative feedback mechanism and thus maintaining pathway activity at an appropriate level in a given cell.

Mutational activation of the Hh pathway, whether sporadic or constitutional as in Gorlin's syndrome, is associated with tumorigenesis in a variety of tissues, but predominantly in skin, the cerebellum and skeletal muscle. The Hh pathway activation, whether triggered by Hh binding (ligand overexpression) or by Ptch mutational inactivation (Ptch is unable to restrain Smo-mediated activation of transcriptional targets through the Gli family even when not bound by the Hh protein), requires Smo regulation (Johnson *et al*, 1996). Smo, which is inactivated by the pathway antagonist cyclopamine, is also a candidate therapeutic target (Incardona *et al*, 1998; Rudin *et al*, 2009; Von Hoff *et al*, 2009).

The role of the Hh pathway in TCC remains poorly studied (Fei *et al*, 2010; Mechlin *et al*, 2010; He *et al*, 2011). The *PTCH1* gene, located in chromosome region 9q22, is a candidate tumour-suppressor gene, as loss of heterozygosity on chromosome arm 9q occurs in more than 50% of TCC (Linnenbach *et al*, 1993; Habuchi *et al*, 1995; Hirao *et al*, 2005), and *PTCH1* mRNA expression is low, compared with normal urothelium, in early-stage tumours exhibiting LOH in the 9q22 region (Aboulkassim *et al*, 2003; Hirao *et al*, 2005). However, few mutations have been detected in the retained *PTCH1* allele (McGarvey *et al*, 1998). Other mechanisms, such as *PTCH1* haploinsufficiency or alterations in other genes regulating the Hh signalling, could be involved in urothelial development. Furthermore, gene amplification of part of chromosome region 12q13-q15, which encompasses *GLI1*, has been found in a subset of bladder cancers (Simon *et al*, 2002). Altered expression of Gli proteins seems to be associated with a more invasive phenotype of bladder tumours *in vitro* (Fei *et al*, 2010; Mechlin *et al*, 2010).

More recently, another mechanism leading to abnormal Hh pathway activation – an autocrine or paracrine loop initiated by Shh overexpression – was detected in gastrointestinal (Berman *et al*, 2003), pancreatic (Thayer *et al*, 2003), and small-cell lung cancer (Watkins *et al*, 2003).

Finally, microRNAs (miRNAs) were recently described as a class of small non-coding cellular RNAs that bind to cis-regulatory elements mainly present in the 3′-untranslated regions (3′-UTRs) of their target-protein-coding mRNAs, resulting in translational regulation (Stefani and Slack, 2008). MicroRNAs are crucial post-transcriptional regulators of gene expression, controlling cell differentiation and proliferation, and being implicated in tumour formation (Calin and Croce, 2006). However, little is known of how miRNAs target specific developmental pathways, including the Hh pathway (Ferretti *et al*, 2008; Uziel *et al*, 2009).

In an attempt to identify new molecular markers in TCC, we analysed a large panel of genes (*n*=31) and miRNAs (*n*=9) involved in the Hh pathway, in a series of 71 urothelial bladder tumours. Using real-time quantitative RT–PCR, we determined expression levels of the selected gene mRNAs and miRNAs in each bladder sample. In this pilot study, the prognostic value of these molecular markers for patient survival was examined retrospectively.

## Patients and methods

### Patients and samples

Normal bladder samples (*n*=5, group I) were obtained during prostatic adenomectomy from patients with no history of bladder cancer.

Bladder cancer samples were obtained from patients who underwent transurethral bladder resection (TURB) or radical cystectomy at Cochin Hospital, Paris, France, between January 2001 and December 2002. All patients signed an informed consent. During TURB, tumour fragments comprising both visible urothelium and underlying muscle were selected for RNA extraction and immediately stored in liquid nitrogen at −80 °C. Remaining fragments were fixed in formaldehyde for pathological analysis. The similar nature of frozen and formaldehyde-fixed samples was confirmed by examining frozen sections of each cryopreserved sample.

Cystectomy specimens were immediately reviewed by the pathologist, who visually selected the tumour zone to be frozen in liquid nitrogen. The rest of the cystectomy specimen was fixed in formaldehyde and subjected to standard pathological analysis after step-sectioning. If insufficient material was available for both nitrogen and formaldehyde storage, the latter took priority.

Each tumour was reviewed by the same pathologist. All the tumours were of urothelial origin. Tumour stage was determined with the 2002 UICC TNM classification of bladder cancer, and tumour grade using the OMS 2004 grading scheme (IUAC, 2002; Molinie, 2006).

There were 11 women and 60 men, with a median age of 68 years (range 42–88 years). Pathological staging showed non-muscle-invasive bladder cancer (NMIBC) in 50 patients (21 low-grade pTa, 10 high-grade pTa, and 19 high-grade pT1) and high-grade muscle-invasive bladder cancer (MIBC, ⩾pT2) in 21 patients.

Outcomes were obtained from the patients’ medical records. After a median follow-up of 71.5 months (range, 1–104), 24 NMIBC patients had recurrences and 4 patients progressed to muscle-invasive disease; statistical analysis was not possible in this group, because of the small number of events. Five NMIBC patients were lost to follow-up and were therefore excluded from the prognostic analysis. Eleven MIBC patients had local or metastatic relapses after a median follow-up of 36.2 months (range, 4–97 months), and all died.

Clinical and histological parameters and outcomes in the NMIBC and MIBC populations are shown in Tables 1a and 1b. These population characteristics are consistent with bladder cancer presentation and evolution.

### Bladder cancer cell lines

We also analysed six bladder cancer cell lines of different origins (CRL1472, CRL1749, CRL2169, HTB2, HTB4, and HTB9) obtained from the American Tissue Type Culture Collection.

### Gene and micro-RNA selection

After examining the literature on the Hh pathway and bladder carcinogenesis, we selected 31 protein-coding genes, including the three Hh pathway ligands (*SHH*, *IHH*, *DHH*), the two homologous Ptch receptors (*PTCH1* and *PTCH2*), transduction and transcription factors, target genes, and 9 miRNAs (Supplementary Data 1 and 2).

### Real-time quantitative RT–PCR analysis of protein-coding genes

The theoretical basis and practical aspects of real-time quantitative RT–PCR (primers and PCR consumables; RNA extraction, cDNA synthesis, and PCR reaction conditions) are described in detail elsewhere (Pignot *et al*, 2009). Quantitative values are obtained from the cycle threshold (Ct), at which the increase in the signal associated with the exponential growth of PCR products begins to be detected. Two endogenous RNA control genes involved in different metabolic pathways were chosen, namely *TBP* (Gen-Bank accession Number NM_003194), which encodes the TATA-box-binding protein, and *RPLP0* (Gen-Bank accession Number NM_001002), which encodes human acidic ribosomal phosphoprotein P0. Each sample was normalised on the basis of its *TBP* (or *RPLP0*) content. Results, expressed as N-fold differences in target gene expression relative to the *TBP* (or *RPLP0*) gene, and termed ‘N*target*’, were determined as N*target*=2^ΔCtsample^, where the ΔCt value of the sample was determined by subtracting the average Ct value of the target gene from the average Ct value of the *TBP* (or *RPLP0*) gene. The N*target* values of the samples were subsequently normalised such that the median of the five normal-bladder N*target* values was 1. For each investigated gene, mRNA values of 3 or more were considered to represent marked overexpression and mRNA values of 0.3 or less were considered to represent marked under-expression. We have previously used the same cutoff points for altered tumour gene expression (Pignot *et al*, 2009). Primers were chosen with the Oligo 6.0 computer programme (National Biosciences, Plymouth, MN, USA). For each primer pair, we performed no-template control and no-RT control (RT negative) assays, which produced negligible signals (Ct values usually >40), suggesting that primer-dimer formation and genomic DNA contamination effects were negligible (Supplementary Data 1). Experiments were performed with duplicates for each data point.

### Real-time quantitative RT–PCR assay of mature miRNAs

MicroRNAs were isolated with the extraction procedure used for the protein-coding genes (total RNA extraction). Reverse transcription was performed with the QIAGEN miScript Reverse Transcription kit, according to the manufacturer's protocol (QIAGEN, GmbH, Hilden, Germany). Specific miRNAs were quantified by real-time PCR with the QIAGEN miScript SYBR Green PCR kit (QIAGEN). The small nucleolar RNA U44 was used as an internal control. The Δ-Δ Ct method was used to determine miRNA expression, as for protein coding gene expression.

### Statistical analysis

Clinical and pathological features of NMIBC and MIBC were tested for their association with tumour recurrence and patient survival, using Student's *t*-test for continuous variables and the *χ*^2^-test for qualitative variables. The distribution of mRNA (or miRNA) levels was analysed using the median and range. Relationships between mRNA (or miRNA) levels and clinical and histological parameters were identified with the Kruskal–Wallis non-parametric *H*-test (link between one qualitative parameter and one quantitative parameter). Overall survival (OS) was calculated from the date of surgery to death from bladder cancer or last follow-up. Survival curves were derived from the Kaplan–Meier estimates. The log-rank test was used to compare survival distributions between subgroups. Differences between two populations were judged significant at confidence levels greater than 95% (*P*<0.05) and all tests were two-sided.

## Results

### mRNA expression in normal bladder tissue

To determine the cutpoint for altered gene expression in tumour samples, the N*target* value of the 31 genes, calculated as described in Patients and Methods, were first determined in 5 normal bladder samples. All the genes had quantifiable mRNA levels by real-time quantitative RT–PCR (Ct value <38), suggesting basal expression of this pathway in normal bladder (Table 2).

The mRNA values were between 0.3 and 3 in normal bladder samples, which helped define values of overexpression and under-expression in tumour samples (mRNA values of 3 or more were considered to represent marked overexpression, and mRNA values of 0.3 or less were considered to represent marked under-expression). We have previously used the same cutoff points for altered tumour gene expression (Pignot *et al*, 2009).

### mRNA expression in bladder tumours according to pathological stage

Table 2 shows mRNA expression levels of the 31 genes relative to the *TBP* endogenous control, according to pathological stage. Similar results were obtained when the endogenous control was *RPLP0*.

#### Ligands

*SHH* showed marked overexpression in bladder cancer compared with normal tissue (*P*<10^−5^), with a median expression level of 78.0 (range 0.22–721.6). The *SHH* overexpression was observed in 96.0% of NMIBC and 52.4% of MIBC, and the median *SHH* mRNA level decreased gradually from pTa low-grade tumours (121.9) to ⩾pT2 tumours (3.92).

#### *IHH and DHH*

expression was less variable, with overexpression in, respectively, 13.5 and 16.9% of tumour samples.

#### Ptch receptors

*PTCH1* and *PTCH2* were under-expressed in, respectively, 31.0 and 43.7% of tumour samples. The *PTCH2* was under-expressed in 56.0% of NMIBC and only 14.3% of MIBC.

#### Transduction factors and regulators

*SMOH* was under-expressed in 74.6% of tumour samples (*P*=0.001).

We observed other markedly under-expressed genes (*P*<0.05) included tumour-suppressor candidates that negatively regulate the Hh pathway (*SUFU*, *GAS1*, *RAB23*) and genes encoding inhibitory proteins (*HHIP*, *HHAT*). The *HHAT* under-expression was far more frequent in MIBC (52.4%) than in NMIBC (2.0%).

Only *DISP2*, a protein-encoding gene involved in the regulation of ligand secretion, was overexpressed (*P*=0.04) both in MIBC (42.9%) and in NMIBC (62.0%).

*DISP1*, *STK36*, *KIF7*, *KIF27*, and *BTRC* expression levels did not differ significantly between tumour samples and normal bladder tissue.

#### Transcription factors (GLI family)

*GLI1* and *GLI2* showed marked under-expression, whereas *GLI3* only tended to be under-expressed. *GLI4*, often described as an antagonistic factor (Kas *et al*, 1996), was overexpressed in 15.5% of tumour samples, but not significantly.

#### Target genes

Five target genes were significantly overexpressed in the tumour samples compared with normal bladder tissue: *FOXM1*, *IGF2*, *OSF2*, and *H19*, which promote cell proliferation and growth, and *SPP1*, which is involved in extracellular matrix interactions. Two of these five genes were either overexpressed or under-expressed, depending on the tumour stage. *IGF2* was overexpressed in NMIBC, and particularly in pTa tumours, whereas it was under-expressed in more than half the MIBC samples. In contrast, *OSF2* was under-expressed in 38% of NMIBC and overexpressed in 57% of MIBC. Two target genes, *PTHR1* and *EPHA7*, were under-expressed, whatever the stage.

### miRNA expression in bladder tumours

Table 3 shows expression levels of the nine selected miRNAs relative to the endogenous control U44, according to pathological stage. Similar results were obtained when two other endogenous miRNA controls (U6B and U48) were used (data not shown).

Six of the nine miRNAs (miRNA-125B, miRNA-326, miRNA-324, miRNA-100, miRNA-361, and miRNA-136) were significantly under-expressed in both NMIBC and MIBC. All these miRNAs have been described as potential inhibitors of the Hh pathway (Tsuda *et al*, 2006; Ferretti *et al*, 2008). Three miRNAs (miRNA-92A, miRNA-19A, and miRNA-20A) were under-expressed in NMIBC, but normally expressed or overexpressed in MIBC, with a gradual increase in expression from pTa low-grade samples to invasive samples. Interestingly, these three miRNA are encoded by the miR-17-92 cluster, a group of miRNA described as oncogenes in several tumours (Northcott *et al*, 2009; Uziel *et al*, 2009).

### *SHH* expression and miRNA expression in bladder cancer cell lines

We also measured the *SHH* mRNA levels in six bladder cancer cell lines: HTB2, HTB4, HTB9, CRL1472, CRL1749, and CRL2169 (Supplementary Data 3). Interestingly, we observed high expression level of *SHH* in cell line HTB2, which is often used as a model system for non-muscle-invasive bladder tumours.

The expression levels of the nine selected miRNA genes were also measured in the six bladder cancer cell lines. High expression levels of miRNA-92A, miRNA-19A, and miRNA-20A and low expression levels of miRNA-125B, miRNA326, miRNA-324, miRNA-100, miRNA-361, and miRNA136 were observed in several cell lines. These results were in keeping with those obtained with the tumour samples. Interestingly, the three miRNAs encoded by the miR-17-92 cluster (miRNA-92A, miRNA-19A, and miRNA-20A) were markedly overexpressed in cell line CRL1472, which is representative of high-grade urothelial bladder carcinoma.

### Correlation between protein-coding mRNA/miRNA expression levels and patient survival

To analyse the expression level as a qualitative variable, the patients were subdivided into equal groups around the median.

#### MIBC

**Figure 1:**
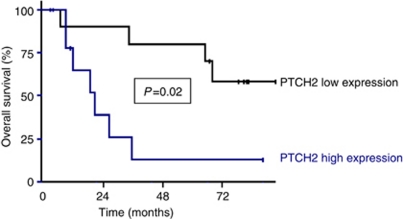
Overall survival curves in MIBC according to expression level of PTCH2.

Among the 20 significantly altered mRNAs, *PTCH2* was the only one with prognostic value in MIBC; high *PTCH2* expression was significantly associated with worse outcome in univariate analysis (*P*=0.02; Figure 1). The 5-year OS rate was 13.0% (s.e.=12.1%) among patients with high *PTCH2* expression and 80.0% (s.e.=12.6%) among patients with low *PTCH2* expression.

**Figure 2:**
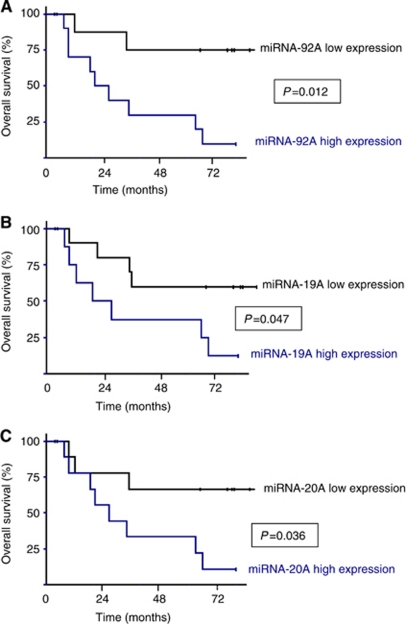
Overall survival curves in MIBC according to expression level of the three miRNA encoded by the miR-17-92 cluster family. (**A**) miRNA-92A, (**B**) miRNA-19A, and (**C**) miRNA-20A.

The expression levels of five of the nine selected miRNA genes were associated with OS among the MIBC patients in univariate analysis. Low miRNA-100 and miRNA-361 expression was significantly associated with better outcome (*P*=0.032 and *P*=0.044, respectively) (Supplementary Data 4). In contrast, high miRNA-92A, miRNA-19A, and miRNA-20A expression was significantly associated with worse outcome (*P*=0.012, *P*=0.047, and *P*=0.036, respectively; Figure 2A–C). The 5-year OS rates were, respectively, 30.0 (s.e.=14.5%), 37.5 (s.e.=17.1%), and 33.3% (s.e.=15.7%) among patients with high miRNA-92A, miRNA-19A, and miRNA-20A expression, *vs* 75.0 (s.e.=15.3%), 60.0 (s.e.=15.5%), and 66.7% (s.e.=15.7%) among patients with low expression.

Multivariate analysis could not be achieved properly due to the pilot nature of this study, which was performed on a small number of patients (only 21 patients with MIBC).

#### NMIBC

Neither mRNA nor miRNA levels were associated with recurrence or progression of NMIBC in univariate analysis.

## Discussion

Several studies suggest that the Hh signalling may contribute to the development of bladder cancer (McGarvey *et al*, 1998; Aboulkassim *et al*, 2003). Here we show that the Hh pathway is activated in TCC, and particularly in NMIBC. *SHH* (encoding the Sonic Hh ligand) and most of the target genes of the Hh pathway were markedly overexpressed, even in pTa low-grade tumours.

It is interesting to note that the expression levels of some *Hh* target genes differ depending on tumour stage (NMIBC or MIBC). For instance, IGF2 is overexpressed in 66% of NMIBC, whereas it is under-expressed in 52.4% of MIBC. At the opposite, OSF2 is under-expressed in 38% of NMIBC and overexpressed in 57.1% of MIBC. This supports the theory that there are two distinct molecular pathways in bladder carcinogenesis, that of hyperplasia and low-grade tumours and/or non-invasive, and that of dysplasia and high-grade tumours and/or infiltrating, with two different gene-expression profile as suggested by Wu (2005).

Constitutive activation of the Hh pathway has been found in several tumour types. In a small subset of the brain, skin and muscle tumours, mutations in *PTCH1* or *SMOH* trigger ligand-independent activation of the Hh pathway (Johnson *et al*, 1996; Cowan *et al*, 1997). Ligand-dependent activation of the Hh pathway has been shown in small-cell lung carcinoma and digestive tract tumours, such as oesophageal carcinoma, gastric carcinoma (Berman *et al*, 2003), and pancreatic carcinoma (Thayer *et al*, 2003; Liu *et al*, 2007). Ligand-dependent oncogenic Hh signalling is associated with high-level expression of the Hh ligand by tumour cells, as observed here with the Sonic Hh ligand. In bladder cancer, Hh pathway activation thus seems to be initiated by overexpression of the Hh ligands, and especially *SHH*, which was markedly overexpressed (>80-fold higher than in normal bladder tissue) both in the bladder tumour samples and in two of the six bladder tumour cell lines tested here. These results are consistent with recent published data, which confirm overexpression of SHH at a protein level (He *et al*, 2011).

As expected (Aboulkassim *et al*, 2003), the expression level of both *PTCH1* and *PTCH2*, which code for the receptors Ptch1 and Ptch2, was lower in tumour samples than in normal tissue, possibly participating in the Hh pathway activation. However, *PTCH2* was re-expressed in MIBC, and this re-expression was associated with poorer OS.

The observed under-expression of *SMOH* and GLI members, associated with significant overexpression of *SHH* ligand and of the majority of the Hh target genes (i.e., *FOXM1, SPP1, IGF2, OSF2, H19*, and *MTSS1*), could be related to the fact that Smo and Gli activity are essentially regulated by post-transcriptional critical events, such as changes in protein conformation, subcellular localisation, phosphorylation, and dimerisation (Denef *et al*, 2000; Taipale *et al*, 2000; Hooper, 2003; Zhu *et al*, 2003) without marked changes at the mRNA level. Alternatively, marked activation of the Hh pathway could lead to a decrease in *SMOH* and *GLI* mRNA levels by a negative feedback.

An additional hypothesis is that tumour cells can produce SHH ligand, stimulating neighbouring stromal cells in paracrine manner, as observed in pancreatic tumours for example (Yauch *et al*, 2008; Bailey *et al*, 2009; Scales and de Sauvage, 2009; Tian *et al*, 2009). Indeed, it may be possible that *SHH* ligand is overexpressed in urothelial tumour cells and that Hh response occurs in supportive stroma. This paracrine signalling could control bladder tumour growth.

Another mechanism potentially regulating the Hh signalling might involve miRNA-mediated post-transcriptional control, a phenomenon recently described in several studies (Tsuda *et al*, 2006; Ferretti *et al*, 2008; Northcott *et al*, 2009; Uziel *et al*, 2009). We obtained evidence that overexpression of a miRNA cluster (miR-17-92) might induce specific activation of the Hh pathway. Overexpression of this cluster has been described in several tumour types and was recently identified as a possible regulator of the Hh pathway. For example, Uziel *et al* (2009) showed that the Hh pathway can be targeted at multiple levels by the same miRNAs in the medulloblastoma. Our findings confirm the existence of a novel regulatory mechanism of Hh signalling in bladder cancer and suggest that misregulation of specific miRNAs may sustain cancer development. Moreover, we found that high expression of 17-92 cluster miRNAs was associated with a poorer vital outcome of MIBC. These results suggest that the Hh pathway activation through overexpression of certain oncogenic miRNAs has prognostic implications, although this needs to be confirmed in a large, independent, and homogenous series of bladder tumours. Indeed, this is a pilot study involving a small number of patients with MIBC, and multivariate analysis (Cox model) could not be performed to confirm the results obtained in univariate analysis.

Moreover, immunochemistry studies are required to confirm these results at a protein level and to precise if the Hh pathway activation effects are epithelial tumour cell specific.

Our finding that the Hh pathway is constitutively activated in bladder tumours raises the possibility of novel therapeutic targets. Indeed, cyclopamine, a plant-derived teratogenic steroidal alkaloid, inhibits the Hh-ligand-dependent and -independent Hh pathway activation by directly interacting with Smo (Incardona *et al*, 1998; Berman *et al*, 2002; Kubo *et al*, 2004). More recently, other drugs, effective *in vitro* and less toxic than cyclopamine (and thus usable in humans), have been described. Two recent clinical trials tested a new drug, GDC-0449, that inhibits the Hh signalling pathway by targeting Smo, in advanced basal-cell carcinoma and medulloblastoma (Rudin *et al*, 2009; Von Hoff *et al*, 2009). Ligand-dependent Hh pathway activation might be blocked by antibodies directly targeting the Sonic Hh ligand or competing for its receptor, as suggested by preclinical studies (Scales and de Sauvage, 2009). However, even if such strategies interfere effectively with the Hh ligand, combination therapy will be needed to deal with other activated oncogenic pathways in the same tumour (Shafaee *et al*, 2006; Olive *et al*, 2009).

The miRNA regulation is another potential mechanism of the Hh pathway inhibition. In a recent study, Tsuda *et al* (2006) showed that a synthetic designer miRNA targeting the 3′-UTRs of Gli-1mRNA effectively inhibited tumour cell proliferation by delaying cell division and activating late apoptosis in pancreatic cell lines.

These recent studies bring compelling evidence that therapies directed against the Hh pathway is a promising new approach for the treatment of several tumours. These new drugs could be useful in the management of non-muscle-invasive bladder tumours, most of which show Hh pathway activation through Shh overexpression, compared with about 50% of muscle-invasive forms. In MIBC, it will be necessary to test tumours for overexpression of the Hh pathway genes, such as the *Sonic Hh ligand* gene, to select patients who are likely to benefit from these drugs.

## Conclusion

We observed constitutive ligand-dependent activation of the Hh pathway in bladder cancer, due to genetic (protein-coding mRNA) and epigenetic (miRNA) dysregulation. The expression levels of *PTCH2* and of miRNAs encoded by the miR-17-92 cluster are attractive candidate prognostic factors in MIBC. Finally, rationalised use of targeted therapies against the Hh pathway could be a new therapeutic hope for selected patients.

## Figures and Tables

**Table 1a:** Clinical and pathological characteristics of the NMIBC subpopulation (*n*=45^a^)

	**No recurrence (*n*=17)**	**Recurrence (*n*=24)**		**Muscle-invasive progression (*n*=4)**
	**Number (%)**	**Number (%)**	***P*-values** b	**Number (%)**
Median age, years (range)	66 (44–86)	68 (42–83)	0.47^c^	78 (77–87)
				
*Sex*			0.36	
Male	13 (76.5)	22 (91.7)		2 (50.0)
Female	4 (23.5)	2 (8.3)		2 (50.0)
				
*Smoking status*			0.56	
Non-smoker	12 (70.6)	20 (83.3)		3 (75.0)
Smoker	5 (29.4)	4 (16.7)		1 (25.0)
				
*History of NMIBC*			**0.001**	
No	15 (88.2)	8 (33.3)		3 (75.0)
Yes	2 (11.8)	16 (66.7)		1 (25.0)
				
*Associated pTis*			0.63	
No	16 (94.1)	23 (95.8)		4 (100)
Yes	1 (5.9)	1 (4.2)		0 (0)
				
*Multifocality*			0.85	
No	11 (64.7)	15 (62.5)		3 (75.0)
Yes	6 (35.3)	9 (37.5)		1 (25.0)
				
*Grade*			0.77	
Low grade	8 (47.1)	9 (37.5)		1 (25.0)
High grade	9 (52.9)	15 (62.5)		3 (75.0)
				
*Tumour stage*			0.93	
Ta	10 (58.8)	15 (62.5)		2 (50.0)
T1	7 (41.2)	9 (37.5)		2 (50.0)

Abbreviations: NMIBC=non-muscle-invasive bladder cancer; pTis=carcinoma *in situ*.

a Five patients lost to follow-up and excluded from the analysis.

b *χ*^2^-test.

c Student's *t*-test.

Bold value indicates significant *P*-value (<0.05).

**Table 1b:** Clinical and pathological characteristics of the MIBC subpopulation (*n*=21)

		**Disease-free survival**	
	**Number of patients (%)**	**Number of events (%)** a	***P*-values** b
*Age*			0.63
<50 years	1 (4.8)	0 (0)	
50–70 years	9 (42.8)	5 (55.6)	
>70 years	11 (52.4)	6 (54.5)	
			
*Sex*			0.15
Male	19 (90.5)	11 (57.9)	
Female	2 (9.5)	0 (0)	
			
*Smoking status*			0.23
Non-smoker	6 (28.6)	4 (66.7)	
Smoker	15 (71.4)	7 (46.7)	
			
*History of NMIBC*			0.27
No	16 (76.2)	7 (43.7)	
Yes	5 (23.8)	4 (80.0)	
			
*Associated pTis*			0.30
No	17 (80.9)	9 (52.9)	
Yes	4 (19.1)	2 (50.0)	
			
*Multifocality*			0.68
No	18 (85.7)	10 (55.6)	
Yes	3 (14.3)	1 (33.3)	
			
*Tumour stage*			**0.0020**
T2	13 (61.9)	5 (38.5)	
>T2	8 (38.1)	6 (75)	
			
*Lymph node status*			**0.0015**
N−	15 (71.4)	5 (33.3)	
N+	6 (28.6)	6 (100)	

Abbreviations: MIBC=muscle-invasive bladder cancer; NMIBC=non-muscle-invasive bladder cancer; pTis=carcinoma *in situ*.

a First recurrence (local or metastatic).

b Log rank test.

Bold values indicate significant *P*-values (<0.05).

**Table 2:** mRNA values of Hedgehog signalling pathway authors

		**NMIBC**						
	**Normal** **Group 1**	**pTaG1–G2** **Group 2**	**pTaG3** **Group 3**	**pT1G3** **Group 4**	**MIBC** **⩾pT2** **Group 5**		**All NMIBC** **2+3+4**	**All tumours** **2+3+4+5**
	***n*=5**	***n*=21**	***n*=10**	***n*=19**	***n*=21**	***P*-values** a	***n*=50**	***n*=71**
*Ligands*								
*SHH* mRNA values: median (range)	1 (0.37–1.81)	121.9 (14.88–566.1)	85.2 (1.16–189.4)	89.9 (9.29–721.6)	3.92 (0.22–293.1)	**<10^−5^**	93.9 (1.16–721.6)	78.0 (0.22–721.6)
Overexpression, *n* (%)	—	21 (100)	8 (80)	19 (100)	11 (52.4)	—	48 (96.0)	59 (83.1)
*IHH* mRNA values: median (range)	1 (0.39–1.53)	0.12 (0.01–2.73)	1.22 (0.08–7.29)	0.32 (0.00–16.3)	0.07 (0.01–1.12)	**0.01**	0.30 (0.00–16.3)	0.23 (0.00–16.3)
Overexpression, *n* (%)	—	0 (0)	2 (20)	5 (26.3)	0 (0)	—	7 (14.0)	7 (13.5)
*DHH* mRNA values: median (range)	1 (0.17–1.92)	1.52 (0.48–3.56)	1.22 (0.40–8.05)	1.83 (0.52–17.9)	1.55 (0.13–5.42)	0.61	1.62 (0.40–17.9)	1.61 (0.13–17.9)
Overexpression, *n* (%)	—	2 (9.5)	2 (20)	3 (15.8)	5 (23.8)	—	7 (14.0)	12 (16.9)
								
*Receptors*								
PTCH1 mRNA values: median (range)	1 (0.82–1.54)	0.61 (0.15–1.59)	0.31 (0.15–1.11)	0.42 (0.07–1.54)	0.39 (0.05–2.19)	0.34	0.47 (0.07–1.59)	0.45 (0.05–2.19)
Under-expression, *n* (%)	—	4 (19)	4 (40)	6 (31.6)	8 (38.1)	—	14 (28.0)	22 (31.0)
PTCH2 mRNA values: median (range)	1 (0.50–1.83)	0.25 (0.00–2.03)	0.39 (0.10–2.76)	0.27 (0.01–1.23)	0.60 (0.06–1.37)	**0.003**	0.26 (0.00–2.76)	0.31 (0.00–2.76)
Under-expression, *n* (%)	—	13 (61.9)	4 (40)	11 (57.9)	3 (14.3)	—	28 (56.0)	31 (43.7)
								
*Transduction factors*								
SMOH mRNA values: median (range)	1 (0.94–1.07)	0.14 (0.02–0.33)	0.13 (0.02–0.42)	0.20 (0.03–0.60)	0.23 (0.02–0.61)	**0.001**	0.15 (0.02–0.60)	0.17 (0.02–0.61)
Under-expression, *n* (%)		19 (90.5)	7 (70)	15 (78.9)	12 (57.1)		41 (82.0)	53 (74.6)
HHIP mRNA values: median (range)	1 (0.68–1.25)	0.26 (0.01–1.48)	0.10 (0.00–0.46)	0.17 (0.01–0.59)	0.03 (0.00–1.22)	**0.0002**	0.18 (0.00–1.48)	0.12 (0.00–1.48)
Under-expression, *n* (%)	—	12 (57.1)	9 (90)	14 (73.7)	20 (95.2)	—	35 (70.0)	55 (77.5)
*SUFU* mRNA values: median (range)	1 (0.18–1.48)	0.30 (0.13–1.45)	0.88 (0.39–4.26)	0.48 (0.14–3.24)	0.29 (0.08–1.39)	**0.01**	0.42 (0.13–4.26)	0.36 (0.08–4.26)
Under-expression, *n* (%)	—	10 (47.6)	0 (0)	6 (31.6)	11 (52.4)	—	16 (32.0)	27 (38.0)
*DISP1* mRNA values: median (range)	1 (0.89–1.33)	0.99 (0.40–2.54)	0.96 (0.46–1.45)	0.86 (0.28–2.95)	0.48 (0.19–1.81)	0.67	0.89 (0.28–2.95)	0.76 (0.19–2.95)
Under-expression, *n* (%)		0 (0)	0 (0)	1 (5.3)	3 (14.3)		1 (2.0)	4 (5.6)
*DISP2* mRNA values: median (range)	1 (0.84–2.57)	3.19 (0.07–23.5)	9.35 (1.17–46.1)	3.40 (0.18–22.0)	2.54 (0.00–25.7)	**0.04**	3.70 (0.07–46.1)	3.42 (0.00–46.1)
Overexpression, *n* (%)	—	11 (52.4)	8 (80)	12 (63.2)	9 (42.9)	—	31 (62.0)	40 (56.3)
*GAS1* mRNA values: median (range)	1 (0.47–1.24)	0.07 (0.03–1.19)	0.12 (0.04–2.35)	0.09 (0.02–0.85)	0.50 (0.03–2.18)	**2 × 10^−5^**	0.09 (0.02–2.35)	0.10 (0.02–2.35)
Under-expression, *n* (%)	—	19 (90.5)	8 (80)	16 (84.2)	8 (38.1)	—	43 (86.0)	51 (71.8)
*STK36* mRNA values: median (range)	1 (0.81–1.59)	1.11 (0.46–2.13)	1.18 (0.54–2.08)	0.75 (0.33–2.51)	0.89 (0.41–3.23)	0.62	0.97 (0.33–2.51)	0.96 (0.33–3.23)
Overexpression, *n* (%)	—	0 (0)	0 (0)	0 (0)	1 (4.8)	—	0 (0)	1 (1.4)
*KIF7* mRNA values: median (range)	1 (0.87–1.48)	0.95 (0.09–2.12)	0.61 (0.15–2.09)	0.67 (0.19–2.86)	0.51 (0.18–1.34)	0.95	0.70 (0.09–2.86)	0.68 (0.09–2.86)
Under-expression, *n* (%)	—	1 (4.8)	1 (10)	1 (5.3)	1 (4.8)	—	3 (6.0)	4 (5.6)
*KIF27* mRNA values: median (range)	1 (0.76–1.26)	0.64 (0.21–2.36)	0.63 (0.25–1.83)	0.63 (0.17–3.34)	0.55 (0.11–3.35)	0.52	0.64 (0.17–3.34)	0.63 (0.11–3.35)
Under-expression, *n* (%)	—	4 (19)	2 (20)	1 (5.3)	2 (9.5)	—	7 (14.0)	9 (12.7)
Overexpression, *n* (%)		0 (0)	0 (0)	1 (5.3)	1 (4.8)		1 (2.0)	2 (2.8)
RAB23 mRNA values: median (range)	1 (0.84–1.24)	0.16 (0.05–0.95)	0.17 (0.07–0.61)	0.11 (0.05–0.75)	0.21 (0.08–1.27)	**2 × 10^−5^**	0.13 (0.05–0.95)	0.16 (0.05–1.27)
Under-expression, *n* (%)	—	20 (95.2)	6 (60)	18 (94.7)	15 (71.4)	—	44 (88.0)	59 (83.1)
*BTRC* mRNA values: median (range)	1 (0.93–1.21)	1.10 (0.40–1.93)	0.91 (0.60–1.77)	0.72 (0.36–1.21)	0.49 (0.25–1.28)	0.62	0.87 (0.36–1.93)	0.74 (0.25–1.93)
Under-expression, *n* (%)	—	0 (0)	0 (0)	0 (0)	1 (4.8)	—	0 (0)	1 (1.4)
								
*Metabolic enzymes*								
*HHAT* mRNA values: median (range)	1 (0.90–1.55)	1.05 (0.47–2.14)	0.99 (0.32–1.61)	0.64 (0.11–2.04)	0.25 (0.04–0.95)	**<10^−5^**	0.95 (0.11–2.14)	0.78 (0.04–2.14)
Under-expression, *n* (%)	—	0 (0)	0 (0)	1 (5.3)	11 (52.4)	—	1 (2.0)	12 (16.9)
								
*Transcription factors*								
GLI mRNA values: median (range)	1 (0.82–1.12)	0.22 (0.03–0.72)	0.18 (0.04–3.58)	0.27 (0.00–1.01)	0.27 (0.01–1.38)	0.048	0.23 (0.00–3.58)	0.24 (0.00–3.58)
Under-expression, *n* (%)	—	15 (71.4)	7 (70)	12 (63.2)	11 (52.4)	—	34 (68.0)	45 (63.4)
GLI2 mRNA values: median (range)	1 (0.47–1.43)	0.08 (0.01–0.61)	0.07 (0.01–1.51)	0.07 (0.02–0.62)	0.21 (0.01–0.68)	**3 × 10^−5^**	0.07 (0.01–1.51)	0.10 (0.01–1.51)
Under-expression, *n* (%)	—	20 (95.2)	7 (70)	18 (94.7)	13 (61.9)	—	46 (92.0)	59 (83.1)
*GLI3* mRNA values: median (range)	1 (0.50–1.31)	0.44 (0.09–1.23)	0.67 (0.18–3.67)	0.28 (0.03–1.99)	0.40 (0.03–1.77)	0.07	0.45 (0.03–3.67)	0.42 (0.03–3.67)
Under-expression, *n* (%)	—	6 (28.6)	2 (20)	11 (57.9)	7 (33.3)	—	19 (38.0)	26 (36.6)
*GLI4* mRNA values: median (range)	1 (0.72–1.48)	1.52 (0.64–4.21)	1.82 (0.62–4.24)	2.00 (0.52–5.15)	1.39 (0.49–4.13)	0.14	1.63 (0.52–5.15)	1.59 (0.49–5.15)
Overexpression, *n* (%)	—	1 (4.8)	3 (30)	5 (26.3)	2 (9.5)	—	9 (18.0)	11 (15.5)
*GLIS1* mRNA values: median (range)	1 (0.62–2.09)	0.01 (0.00–0.15)	0.01 (0.00–0.20)	0.02 (0.00–0.07)	0.08 (0.00–0.27)	**<10^−5^**	0.01 (0.00–0.20)	0.02 (0.00–0.27)
Under-expression, *n* (%)	—	21 (100)	10 (100)	19 (100)	21 (100)	—	50 (100)	71 (100)
*GLIS2* mRNA values: median (range)	1 (0.83–1.10)	0.17 (0.04–0.59)	0.26 (0.07–0.87)	0.28 (0.09–0.74)	0.68 (0.04–2.02)	**0.0002**	0.22 (0.04–0.87)	0.27 (0.04–2.02)
Under-expression, *n* (%)	—	18 (85.7)	5 (50)	11 (57.9)	5 (23.8)	—	34 (68.0)	39 (54.9)
								
*Target genes*								
*FOXM1* mRNA values: median (range)	1 (0.62–1.14)	3.42 (0.74–26.8)	12.2 (6.94–23.0)	19.9 (6.21–82.4)	37.4 (11.84–98.7)	**<10^−5^**	13.4 (0.74–82.4)	17.8 (0.74–98.7)
Overexpression, *n* (%)	—	13 (61.9)	10 (100)	19 (100)	21 (100)	—	42 (84.0)	63 (88.7)
*SPP1* mRNA values: median (range)	1 (0.85–2.51)	1.16 (0.06–14.0)	1.62 (0.60–13.1)	2.87 (0.21–214.5)	5.54 (0.44–43.0)	**0.02**	1.65 (0.06–214.5)	2.58 (0.06–214.5)
Overexpression, *n* (%)	—	5 (23.8)	3 (30)	10 (52.6)	13 (61.9)	—	18 (36.0)	31 (43.7)
IGF2 mRNA values: median (range)	1 (0.70–2.37)	44.0 (0.07–209.4)	54.5 (7.09–217.5)	1.02 (0.05–232.3)	0.26 (0.04–13.8)	**<10^−5^**	11.5 (0.05–232.3)	2.93 (0.04–232.3)
Under-expression, *n* (%)	—	3 (14.3)	0 (0)	5 (26.3)	11 (52.4)	—	8 (16.0)	19 (26.8)
Overexpression, *n* (%)	—	16 (76.2)	10 (100)	7 (36.8)	2 (9.5)	—	33 (66.0)	35 (49.3)
*OSF2* mRNA values: median (range)	1 (0.64–1.10)	0.45 (0.08–5.19)	0.45 (0.01–19.0)	0.55 (0.01–7.31)	3.43 (0.13–39.3)	**0.0005**	0.52 (0.01–19.0)	0.77 (0.01–39.3)
Under-expression, *n* (%)	—	9 (42.9)	4 (40)	6 (31.6)	3 (14.3)	—	19 (38.0)	22 (31.0)
Overexpression, *n* (%)	—	1 (4.8)	1 (10)	2 (10.5)	12 (57.1)	—	4 (8.0)	16 (22.5)
*EPHA7* mRNA values: median (range)	1 (0.49–1.46)	0.12 (0.00–0.77)	0.08 (0.05–1.25)	0.11 (0.00–0.59)	0.07 (0.00–0.79)	**0.0008**	0.10 (0.00–1.25)	0.09 (0.00–1.25)
Under-expression, *n* (%)	—	19 (90.5)	7 (70)	16 (84.2)	15 (71.4)	—	42 (84.0)	57 (80.3)
*PTHR1* mRNA values: median (range)	1 (0.98–1.54)	0.14 (0.03–0.76)	0.13 (0.04–0.50)	0.10 (0.00–0.37)	0.08 (0.01–0.55)	**4 × 10^−5^**	0.12 (0.00–0.76)	0.11 (0.00–0.76)
Under-expression, *n* (%)	—	19 (90.5)	8 (80)	18 (94.7)	17 (81.0)	—	45 (90.0)	62 (87.3)
*H19* mRNA values: median (range)	1 (0.85–2.12)	16.9 (0.03–196.7)	10.9 (0.89–186.1)	0.76 (0.02–105.8)	2.38 (0.31–101.8)	**0.02**	6.74 (0.02–196.7)	3.35 (0.02–196.7)
Overexpression, *n* (%)	—	14 (66.7)	7 (70)	7 (36.8)	8 (38.1)	—	28 (56.0)	36 (50.7)
MTSS1 mRNA values: median (range)	1 (0.82–1.62)	2.01 (0.64–5.15)	3.39 (1.28–7.49)	1.83 (0.38–6.28)	1.65 (0.47–5.28)	0.21	2.00 (0.38–7.49)	1.91 (0.38–7.49)
Overexpression, *n* (%)	—	8 (38.1)	5 (50)	6 (31.6)	4 (19.0)	—	19 (38.0)	23 (32.4)

Abbreviations: DHH=Desert Hedgehog; IHH=Indian Hedgehog; MIBC=muscle-invasive bladder cancer; NMIBC=non-muscle-invasive bladder cancer; SHH=Sonic Hedgehog.

a *χ*^2^-test (comparison of under- or overexpression in group 1 *vs* group 2 *vs* group 3 *vs* group 4 *vs* group 5).

Bold values indicate significant *P*-values (<0.05).

**Table 3:** Values of the nine miRNAs involved in the Hedgehog signalling pathway

		**NMIBC**			**MIBC**			
	**Normal**	**pTaG1-G2**	**pTaG3**	**pT1G3**	**⩾pT2**		**All NMIBC**	**All tumours**
	**Group 1**	**Group 2**	**Group 3**	**Group 4**	**Group 5**		**2+3+4**	**2+3+4+5**
	***n*=5**	***n*=21**	***n*=10**	***n*=19**	***n*=21**	***P*-values** a	***n*=50**	***n*=71**
125B miRNA values: median (range)	1 (0.71–2.33)	0.01 (0.00–0.29)	0.00 (0.00–0.22)	0.02 (0.00–0.37)	0.09 (0.01–1.52)	**<10^−5^**	0.01 (0.00–0.37)	0.03 (0.00–1.52)
Under-expression, *n* (%)	—	21 (100)	10 (100)	17 (89.5)	17 (81.0)	—	48 (96.0)	65 (91.5)
326 miRNA values: median (range)	1 (0.59–1.49)	0.02 (0.01–0.23)	0.02 (0.01–0.12)	0.04 (0.01–0.33)	0.08 (0.03–3.13)	**<10^−5^**	0.03 (0.01–0.33)	0.04 (0.01–3.13)
Under-expression, *n* (%)	—	21 (100)	10 (100)	17 (89.5)	19 (90.5)	—	48 (96.0)	67 (94.4)
324 miRNA values: median (range)	1 (0.59–2.51)	0.15 (0.03–0.98)	0.15 (0.05–0.46)	0.26 (0.04–1.69)	0.32 (0.05–1.38)	**0.001**	0.22 (0.03–1.69)	0.23 (0.03–1.69)
Under-expression, *n* (%)	—	18 (85.7)	8 (80)	13 (68.4)	9 (42.9)	—	39 (78.0)	48 (67.6)
100 miRNA values: median (range)	1 (0.36–2.09)	0.01 (0.00–0.20)	0.00 (0.00–0.06)	0.01 (0.00–0.30)	0.03 (0.00–0.81)	**<10^−5^**	0.01 (0.00–0.30)	0.01 (0.00–0.81)
Under-expression, *n* (%)	—	21 (100)	10 (100)	19 (100)	19 (90.5)	—	50 (100)	69 (97.2)
361 miRNA values: median (range)	1 (0.60–2.38)	0.19 (0.08–0.98)	0.16 (0.13–0.30)	0.32 (0.07–1.59)	0.36 (0.06–1.13)	**0.0001**	0.24 (0.07–1.59)	0.29 (0.06–1.59)
Under-expression, *n* (%)	—	15 (71.4)	10 (100)	8 (42.1)	6 (28.6)	—	33 (66.0)	39 (54.9)
136 miRNA value: median (range)	1 (0.36–2.23)	0.03 (0.02–0.34)	0.02 (0.02–0.18)	0.06 (0.01–0.50)	0.12 (0.04–4.68)	**1 × 10^−5^**	0.05 (0.01–0.50)	0.06 (0.01–4.68)
Under-expression, *n* (%)	—	19 (90.5)	10 (100)	17 (89.5)	17 (81.0)	—	46 (92.0)	63 (88.7)
92A miRNA values: median (range)	1 (0.72–2.22)	0.29 (0.10–1.94)	0.33 (0.14–1.02)	0.63 (0.12–5.66)	1.44 (0.33–5.67)	**0.003**	0.41 (0.10–5.66)	0.54 (0.10–5.67)
Under-expression, *n* (%)	—	11 (52.4)	2 (20)	3 (15.8)	0 (0)	—	16 (32.0)	16 (22.5)
Overexpression, *n* (%)	—	0 (0)	0 (0)	1 (5.3)	4 (19.0)	—	1 (2.0)	5 (7.0)
19A miRNA values: median (range)	1 (0.68–1.46)	0.22 (0.03–0.65)	0.31 (0.12–0.82)	0.52 (0.13–2.12)	0.74 (0.11–9.21)	**<10^−5^**	0.32 (0.03–2.12)	0.48 (0.03–9.21)
Under-expression, *n* (%)	—	17 (81.0)	4 (40)	4 (21.1)	2 (9.5)	—	25 (50.0)	27 (38.0)
Overexpression, *n* (%)	—	0 (0)	0 (0)	0 (0)	5 (23.8)	—	0 (0)	5 (7.0)
20A miRNA values: median (range)	1 ([0.64–1.67)	0.13 (0.05–1.03)	0.17 (0.11–0.46)	0.37 (0.09–2.34)	0.82 (0.14–2.98)	**<10^−5^**	0.26 (0.05–2.34)	0.30 (0.05–2.98)
Under-expression, *n* (%)	—	18 (85.7)	8 (80)	8 (42.1)	3 (14.3)	—	34 (68.0)	37 (52.1)

Abbreviations: MIBC=muscle-invasive bladder cancer; NMIBC=non-muscle-invasive bladder cancer.

a *χ*^2^-test (comparison of under- or overexpression in group 1 *vs* group 2 *vs* group 3 *vs* group 4 *vs* group 5).

Bold values indicate significant *P*-values (<0.05).
