# Supplementary material for: Hedgehog pathway activation in human transitional cell carcinoma of the bladder
Source: Br J Cancer. 2012 Feb 23;106(6):1177–86. doi: 10.1038/bjc.2012.55 (PMC3304423; doi:10.1038/bjc.2012.55)
Supplement: Supplementary Data 1 [file bjc201255x1.doc]

**Supplemental data 1: Protein-coding genes involved in the Hedgehog signaling pathway and oligonucleotide primer sequences used**

|  | Symbols | **Alternative Symbols** | Definition | Primers **(primer forward – primer reverse)** |
| --- | --- | --- | --- | --- |
| **Ligands** | | | | |
|  | *SHH* | *HHG1* | Sonic hedgehog | ^5'^ CCGGCTTCGACTGGGTGTACTA ^3'^ – ^5'^ CGCCACCGAGTTCTCTGCTTT ^3'^ |
|  | *IHH* | *HHG2* | Indian hedgehog | ^5'^ AGGCCGGCTTTGACTGGGTGTATT ^3'^ – ^5'^ GCGGCCGAGTGCTCGGACTT ^3'^ |
|  | *DHH* | *HHG3* | Desert hedgehog | ^5'^ CCGGCTTCGACTGGGTCTACTAC ^3'^ – ^5'^ GACCGCCAGTGAGTTATCAGCTTT ^3'^ |
| **Receptors** | | | | |
|  | *PTCH1* | *PTC1* | Patched 1 | ^5'^ CCCCTGTACGAAGTGGACACTCTC ^3'^ – ^5'^ AAGGAAGATCACCACTACCTTGGCT ^3'^ |
|  | *PTCH2* | *PTC2* | Patched 2 | ^5'^ GATGGGGCCATCTCCACATT ^3'^ – ^5'^ CGCCGCAAAGAAGTACCTTACA ^3'^ |
| **Transduction factors** | | | | |
|  | *SMOH* | *SMO* | Smoothened homolog | ^5'^ GCTACTTCCTCATCCGAGGAGTCA ^3'^ – ^5'^ GGCGCAGCATGGTCTCGTT ^3'^ |
|  | *HHIP* | *HIP* | Hedgehog interacting protein | ^5'^ GGGCGCCTGGAGAATAAGATATTT ^3'^ - ^5'^ GTGGAGAGCAAAGTGCACATTTGA ^3'^ |
|  | *SUFU* | *SUFUH* | Suppressor of fused homolog | ^5'^ GCTGCTGACAGAGGACCCACA ^3'^ - ^5'^ GTGCAGACACCAACGATCTGGA ^3'^ |
|  | *DISP1* | *DISPA* | Dispatched homolog 1 | ^5'^ ACTTCTCTGATCCATTGCTGGGTT ^3'^ - ^5'^ GACCAATCTCTGGCCTATTGCTGT ^3'^ |
|  | *DISP2* | *DISPB* | Dispatched homolog 2 | ^5'^ TGTGCAGCACCATGTGGTCA ^3'^ - ^5'^ AGCAATCAGCTGGGAATAGCTCTT ^3'^ |
|  | *GAS1* | *GAS-1* | Growth arrest-specific 1 | ^5'^ CGTCATTGAGGACATGCTGGCTAT ^3'^ - ^5'^ TTCTCCTTGACCGACTCGCAGAT ^3'^ |
|  | *STK36* | *FU* | Serine/threonine kinase 36 | ^5'^ ACCCCAGATTGTGAACGAGCAT ^3'^ - ^5'^ CATTGTCACTGTCTGGCTCCTCAT ^3'^ |
|  | *KIF7* |  | Kinesin family member 7 | ^5'^ GTCCCAGTGCGAGATGAACCT ^3'^ - ^5'^ CGGAGCGTCACCACCTTGT ^3'^ |
|  | *KIF27* |  | Kinesin family member 27 | ^5'^ AGCTTGCCTGAGTCCTGTTGAGATTA ^3'^ - ^5'^ GCTTCTCGCAAATTCACCACCTTA ^3'^ |
|  | *RAB 23* | *HSPC137* | Ras-related protein Rab-23 | ^5'^ GAAAGTAGTAGCCGAAGTGGGAGAT ^3'^ - ^5'^ AGTGCCTCAGCTTCCTCATTCTT ^3'^ |
|  | *BTRC* | *FBW1A* | Beta-transducin repeat containing | ^5'^ GTCTACGGACCCTTGTGGAGCAT ^3'^ - ^5'^ GGGCAGCTGGATCATTTAGGAAGT ^3'^ |
| **Metabolic enzymes** | | | | |
|  | *HHAT* | *SKI1* | Hedgehog acyltransferase | ^5'^ CCTGGATGCTGGCCTATGTCTT ^3'^ - ^5'^ GCTCCTGCTGCTGCATCTGTT ^3'^ |
| **Transcription factors** | | | | |
|  | *GLI1* | *GLI* | Glioma-associated oncogene family zinc finger 1 | ^5'^ CCAACTCCACAGGCATACAGGAT ^3'^ - ^5'^ CACAGATTCAGGCTCACGCTTC ^3'^ |
|  | *GLI2* | *HPE9* | Glioma-associated oncogene family zinc finger 2 | ^5'^ AAGTCACTCAAGGATTCCTGCTCA ^3'^ - ^5'^ GTTTTCCAGGATGGAGCCACTT ^3'^ |
|  | *GLI3* | *PAPA* | Glioma-associated oncogene family zinc finger 3 | ^5'^ CGCGACTGAACCCCATTCTAC ^3'^ - ^5'^ GTGTTGTTGGACTGTGTGCCATT ^3'^ |
|  | *GLI4* | *HKR4* | Glioma-associated oncogene family zinc finger 4 | ^5'^ CCATGGGCATCAACATGGCT ^3'^ - ^5'^ TCCTCTACGTCTTGGAGATCCAGGT ^3'^ |
|  | *GLIS1* | *FLJ36155* | GLI-similar 1 | ^5'^ CCCAGCCCACAAGGTTACCA ^3'^ - ^5'^ CATCCGGTAGCAGTCGCCATA ^3'^ |
|  | *GLIS2* | *FLJ38247* | GLI-similar 2 | ^5'^ GTGTCGCTGGGCCAAGTGTAA ^3'^ - ^5'^ CGGGCTTGACATGGTAATCGTT ^3'^ |
| **Target genes** | | | | |
|  | *FOXM1* | *MPP2* | Forkhead box M1 | ^5'^ GGGAGACCTGTGCAGATGGTGA ^3'^ - ^5'^ TCGAAGCCACTGGATGTTGGAT ^3'^ |
|  | *SPP1* | *OPN* | Secreted phosphoprotein 1 | ^5'^ TCGCAGACCTGACATCCAGTACC ^3'^ - ^5'^ CCATTCAACTCCTCGCTTTCCAT ^3'^ |
|  | *IGF2* | *FLJ44734* | Insulin-like growth factor 2 | ^5'^ CGACCGTGCTTCCGGACAAC ^3'^ - ^5'^ AGGCGCTGGGTGGACTGCTT ^3'^ |
|  | *OSF-2* | *POSTN* | Osteoblast specific factor 2 | ^5'^ GTCCTAATTCCTGATTCTGCCAAA ^3'^ - ^5'^ GGGCCACAAGATCCGTGAA ^3'^ |
|  | *EPHA7* | *HEK11* | Ephrin type-A receptor 7 | ^5'^ GCAGGAACCAGAGCATCCCA ^3'^ - ^5'^ GGTCCGTTCCCTTTGATCTTTCT ^3'^ |
|  | *PTHR1* | *PTHR* | Parathyroid hormone 1 receptor | ^5'^ ACAACAGGACGTGGGCCAACTAC ^3'^ - ^5'^ CGGTCAAACACCTCCCGTTCA ^3'^ |
|  | *H19* | *WT2* | H19, imprinted maternally expressed untranslated mRNA | ^5'^ TCCTCCCACGGAGTCGGCA ^3'^ - ^5'^ AGCTGGGTAGCACCATTTCTTTCA ^3'^ |
|  | *MTSS1* | *MIM* | Metastasis suppressor 1 | ^5'^ GCAGTCCCAGCTTCGGACAA ^3'^ - ^5'^ GGTGGCCATGTCAGCCACTT ^3'^ |
