# Supplementary material for: Hedgehog pathway activation in human transitional cell carcinoma of the bladder
Source: Br J Cancer. 2012 Feb 23;106(6):1177–86. doi: 10.1038/bjc.2012.55 (PMC3304423; doi:10.1038/bjc.2012.55)
Supplement: Supplementary Data 2 [file bjc201255x2.doc]

**Supplemental data 2: MicroRNAs involved in the Hedgehog signaling pathway**

| **Official symbol** | **miRNA name** | **Target gene** | **Tumor suppressor gene / oncogene** | **Reference** |
| --- | --- | --- | --- | --- |
| **MIRN125B** | Hs_miR-125b_1 | SMOH | suppressor | Ferretti E *et al*., EMBO J, 2008 |
| **MIRN326** | Hs_miR-326_1 | SMOH | suppressor | Ferretti E *et al*., EMBO J, 2008 |
| **MIRN324** | Hs_miR-324-5p_1 | SMOH + GLI1 | suppressor | Ferretti E *et al*., EMBO J, 2008 |
| **MIRN100** | Hs_miR-100_1 | GLI1 | suppressor | Ferretti E *et al*., EMBO J, 2008 |
| **MIRN361** | Hs_miR-361_1 | GLI1 | suppressor | Tsuda N *et al*., Clin Cancer Res, 2006 |
| **MIRN136** | Hs_miR-136_1 | GLI1 | suppressor | Tsuda N *et al*., Clin Cancer Res, 2006 |
| **MIRN92A** | Hs_miR-92a_1 | unknown | oncogene | Uziel T *et al*., Proc Natl Acad Sci USA, 2009 |
| **MIRN19A** | Hs_miR-19a_1 | unknown | oncogene | Uziel T *et al*., Proc Natl Acad Sci USA, 2009 |
| **MIRN20A** | Hs_miR-20a_1 | unknown | oncogene | Uziel T *et al*., Proc Natl Acad Sci USA, 2009 |
