# Supplementary material for: Hedgehog pathway activation in human transitional cell carcinoma of the bladder
Source: Br J Cancer. 2012 Feb 23;106(6):1177–86. doi: 10.1038/bjc.2012.55 (PMC3304423; doi:10.1038/bjc.2012.55)
Supplement: Supplementary Data 3 [file bjc201255x3.doc]

# Supplemental data 3: *SHH* and miRNA expression in bladder cancer cell lines

|  |  |  |  |  |  |  |  | | |
| --- | --- | --- | --- | --- | --- | --- | --- | --- | --- |
|  | **CRL1472** | **CRL1749** | **CRL2169** | **HTB2** | **HTB4** | **HTB9** | **Normal Bladder**  **(n=5)** | **NMIBC (n=50)** | **MIBC (n=21)** |
|  |  |  |  |  |  |  |  |  |  |
| ***SHH*** | NQ | 6.20 | NQ | **219** | 2.88 | 17.2 | 5.54 | **520** | 21.7 |
|  |  |  |  |  |  |  |  |  |  |
| *miR-****125B*** | 20638 | 146407 | 10561 | 219 | 140953 | 51578 | 202565 | 2026 | 18231 |
|  |  |  |  |  |  |  |  |  |  |
| *miR-****326*** | 4034 | 480 | 2617 | 1052 | 1016 | 1133 | 2059 | 61.8 | 165 |
|  |  |  |  |  |  |  |  |  |  |
| *miR-****324*** | 7817 | 1364 | 2605 | 2482 | 2279 | 914 | 5812 | 1279 | 465 |
|  |  |  |  |  |  |  |  |  |  |
| *miR-****100*** | 12383 | 138233 | 5921 | 290 | 69593 | 7743 | 66058 | 661 | 1982 |
|  |  |  |  |  |  |  |  |  |  |
| *miR-****361*** | 1198 | 185 | 258 | 483 | 742 | 284 | 1252 | 300 | 451 |
|  |  |  |  |  |  |  |  |  |  |
| *miR-****136*** | 2.94 | 2.36 | NQ | 1.16 | 2.19 | NQ | 962 | 48.1 | 115 |
|  |  |  |  |  |  |  |  |  |  |
| *miR-****92A*** | **207829** | 73099 | 55619 | 58389 | 78748 | 140563 | 43086 | 17665 | 62044 |
|  |  |  |  |  |  |  |  |  |  |
| *miR-****19A*** | **33344** | 4071 | 6035 | 2265 | 13792 | 14069 | 10431 | 3338 | 7719 |
|  |  |  |  |  |  |  |  |  |  |
| *miR-****20A*** | **117233** | 17405 | 19835 | 17323 | 44030 | 62979 | 44913 | 11677 | 36829 |
|  |  |  |  |  |  |  |  |  |  |
|  |  |  |  |  |  |  |  |  |  |

Expression levels were normalized such that the value for the “basal expression level” (smallest amount of *SHH* mRNA and target microRNA quantifiable, Ct=35) was 1.

Median expression values in normal and tumor bladder samples are given for information.

NQ, not quantifiable: very low levels of target mRNA or microRNA that were only detectable but not quantifiable by means of the real-time quantitative RT-PCR assay (Ct values > 35).
