# Supplementary material for: Hedgehog pathway activation in human transitional cell carcinoma of the bladder
Source: Br J Cancer. 2012 Feb 23;106(6):1177–86. doi: 10.1038/bjc.2012.55 (PMC3304423; doi:10.1038/bjc.2012.55)
Supplement: Supplementary Data 4 [file bjc201255x4.doc]

**Supplemental data 4: Overall survival curves in MIBC according to expression level of miRNA 100 and miRNA 361**

**S4a: Overall survival curves in MIBC according to expression level of miRNA 100**

**
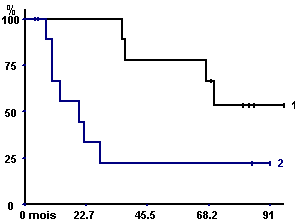
**

**Overall survival (%)**

miRNA 100 low expression

miRNA 100 high expression

**p=0.032**

**0 24 48 72 90**

**Time (months)**

**S4b: Overall survival curves in MIBC according to expression level of miRNA 361**

**
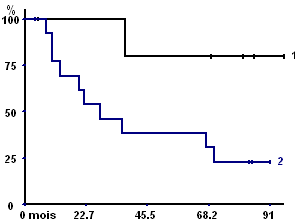
**

**Overall survival (%)**

miRNA 361 low expression

miRNA 361 high expression

**p=0.044**

**0 24 48 72 90**

**Time (months)**
